# Isothiocyanates induce autophagy and inhibit protein synthesis in primary cells *via* modulation of AMPK-mTORC1-S6K1 signaling pathway, and protect against mutant huntingtin aggregation

**DOI:** 10.1007/s00394-024-03539-z

**Published:** 2024-12-16

**Authors:** Joanna Brokowska, Anna Herman-Antosiewicz, Aleksandra Hać

**Affiliations:** 1 Department of Medical Biology and Genetics, Faculty of Biology, University of Gdansk, Wita Stwosza 59, Gdansk, 80-308 Poland; 2 Present Address: Department of Molecular Biology, Faculty of Biology, University of Gdansk, Gdansk, Poland

**Keywords:** Primary cells, Autophagy, Isothiocyanates, AMPK, Mutant huntingtin (mHtt), Huntington’s disease (HD)

## Abstract

**Purpose:**

Autophagy is a degradation process whose activation underlies beneficial effects of caloric restriction. Isothiocyanates (ITCs) induce autophagy in cancer cells, however, their impact on primary cells remains insufficiently explored, particularly in non-epithelial cells. The aim of this study was to investigate whether ITCs induce autophagy in primary (non-immortalized) mesenchymal cells and if so, to determine the molecular mechanism underlying its activation and consequences on cell functioning.

**Methods:**

Primary human dermal fibroblasts (HDFa) and prostate cancer cells (PC3) as well as two ITCs, sulforaphane and phenethyl isothiocyanate, were applied. Cell viability was measured by the MTT test, protein synthesis - by ^3^H-leucine incorporation, and protein level - by immunoblotting. A number of mutant huntingtin (mHtt) aggregates was assessed by fluorescence microscopy.

**Results:**

Both ITCs efficiently induced autophagy in fibroblasts which coincided with suppression of mTORC1 – a negative autophagy regulator - and protein synthesis arrest. A dephosphorylation of mTORC1 substrate, S6K1, and ribosomal S6 protein was preceded by activation of AMPK, an inhibitor of mTORC1 and autophagy activator. A similar response was observed in phenethyl isothiocyanate-treated prostate cancer cells. We also showed that ITCs-induced autophagy and/or translation block do not affect cells viability and can protect cells against an accumulation of mHtt aggregates – a main cause of Huntington’s disease.

**Conclusion:**

Our study showed that ITCs induce autophagy and inhibit protein synthesis in both primary mesenchymal and cancer cells *via* modulation of the AMPK-mTORC1-S6K1 pathway. Moreover, it suggests that ITCs might have a potential in developing therapeutics for Huntington’s disease.

**Supplementary Information:**

The online version contains supplementary material available at 10.1007/s00394-024-03539-z.

## Introduction

Macroautophagy (herein referred to as autophagy) is an evolutionarily conserved process enabling lysosomal degradation of bulk cytoplasmic contents, long-lived proteins and whole organelles in eukaryotic cells. Such components are first engulfed by an isolation membrane and sequestered into double-membrane organelles – autophagosomes - which in the next step are transported along acetylated microtubules to deliver their contents to lysosomes for degradation. The lipidated form of LC3 (LC3-II), which is conjugated to the autophagosome membrane upon autophagy induction, is used as the marker of the process [1].

Autophagy is intimately connected with cellular homeostasis and plays a key role during stress conditions contributing to cell survival and its deregulation is associated with pathological processes including neurodegeneration, myopathies and cancer [2–7]. It enables a cell to eliminate damaged and thus potentially harmful organelles and protein aggregates as well as provides substrates necessary for maintaining the metabolic processes under starvation [7–9]. Autophagy stimulation is considered to underlie the beneficial effects of caloric restriction and exercise on human health and the improvement in metabolic conditions in obese individuals. At the molecular level, upregulation of autophagy by caloric restriction or exercises occurs via activation of AMP-activated protein kinase (AMPK) [10–12].

AMPK, mTORC1 (mechanistic target of rapamycin complex 1) and Ulk1/2 (UNC-51-like kinase-1 and 2) are critical components in the regulation of mammalian autophagy, mutually modulating each other’s activity. AMPK, a key energy sensor that regulates cellular metabolism to maintain energy homeostasis, promotes autophagy. Conversely, it is inhibited by mTORC1, a master growth regulator that senses and integrates signals of nutrients and growth factors availability and promotes, among others, protein synthesis by phosphorylation of its substrate, ribosomal S6 protein kinase 1 (S6K1). Under stress conditions, AMPK stimulates autophagy by activation of Ulk1 and suppression of mTORC1 which consequently results in a block in protein synthesis [13, 14].

In addition to nutrient deprivation, pathogen infection and formation of protein aggregates, autophagy can be induced by various natural compounds. Studies performed on prostate and breast cancer cells demonstrated autophagy-inducing properties of isothiocyanates (ITCs) – natural compounds present in a human diet in edible vegetables, such as broccoli, Brussels sprouts or cauliflower [9, 15]. Chemopreventive and anticancer activity of ITCs was confirmed by epidemiological data, experiments with chemically induced cancers in animals or in vitro experiments performed on cancer cell lines [16, 17]. They were also shown to be safe and cause no side effects in animal models [18]. Despite extensive studies, the molecular mechanism of their activity, including autophagy induction, is still not fully elucidated [9, 15]. The vast majority of research investigating ITCs was performed on cancer cell lines. However, since malignant cells possess severe abnormalities in basic cellular processes, their response might differ and be unrepresentative compared to healthy ones. On the other hand, the impact of ITCs on primary - non-cancerous and non-immortalized - cells, including cells of non-epithelial origin, is much less explored, and such knowledge might be particularly important in the context of potential future application of ITCs as human therapeutics.

The present study was designed to investigate whether the induction of autophagy by ITCs is limited to neoplastic cells or is a general mechanism occurring in primary cells as well, and if so, to identify its molecular basis and consequences exerted on cell functioning.

## Methods

### Reagents

D, L-sulforaphane (SFN; purity 99%) and phenethyl isothiocyanate (PEITC; purity 98%) were purchased from LKT Laboratories (St. Paul, MN, USA). Tissue culture media, penicillin/streptomycin antibiotic mixture and fetal bovine serum were obtained from Thermo Fisher Scientific (Waltham, MA, USA). Plasmid pEGFP-Q74 encoding the first exon of mutant huntingtin protein (mHtt) with 74 polyglutamine repeats and fused with GFP was a gift from David Rubinsztein (Addgene plasmid #40262; http://n2t.net/addgene:40262; RRID: Addgene_40262) (3). Transfection with plasmid DNA was performed using Viromer Yellow transfection reagent (Lipocalyx GmbH, Halle, Germany). The antibodies against p-S6 (Ser-235; sc-101793), p-S6K1 (Thr-389; sc-11759) and GFP (sc-9996) were from Santa Cruz Biotechnology (Santa Cruz, CA), antibodies against PARP (#9542), p-AMPK (Thr-172; 40H9) and AMPK (#2793) were from Cell Signaling Technology (Danvers, MA, USA), antibodies against LC3 (PM036) - from MBL International (Woburn, MA, USA) and antibodies against S6K1 - from Merck Millipore (Burlington, MA, USA). The anti-β-actin (A3854), anti-mouse (A9169) and anti-rabbit (A9044) antibodies conjugated with HRP, as well as DMSO and Thiazolyl Blue Tetrazolium Bromide (MTT) were from Sigma–Aldrich (St. Louis, MO, USA). Protease and phosphatase inhibitor cocktails were obtained from Roche Diagnostics (Basel, Switzerland). L-[3, 4, 5-^3^H]-leucine was purchased from Perkin Elmer (Waltham, MA, USA).

### Cell culture

Human dermal adult fibroblasts (HDFa) were obtained from Thermo Fisher Scientific (Product Line Cascade Biologics™) and were maintained in Dulbecco’s Modified Eagle Medium (high glucose) supplemented with 10% (v/v) heat-inactivated fetal bovine serum and penicillin-streptomycin antibiotic mixture. Prostate cancer PC3 cells were kindly provided by prof. Danuta Duś (Ludwik Hirszfeld Institute of Immunology and Experimental Therapy, Polish Academy of Sciences) and were maintained in F12-K Nutrient Mixture medium supplemented with 9% (v/v) fetal bovine serum and penicillin-streptomycin antibiotic mixture. Each cell line was maintained at 37^°^C in a humidified atmosphere of 95% air and 5% CO_2_. D, L-sulforaphane and phenethyl isothiocyanate were prepared in DMSO at a stock concentration of 2,5–40 mM and stored at ­20^°^C. Control cells were treated with an equal amount of pure DMSO (final concentration 0.1%).

### Viability assay

Cell viability was determined by the MTT test. Cells were seeded at a density of 4 × 10^3^ per well at a 96-well plate in triplicate and allowed to attach overnight. The medium was replaced with a fresh one supplemented with DMSO (control; 0.1% v/v) or indicated concentration of tested ITCs for 24 h. Next, thiazolyl blue tetrazolium bromide (MTT) solution was added to wells to a final concentration of 1 mg/ml for 3 h. After the end of the incubation, the medium was removed and crystals at the bottom of the wells were dissolved in 100% DMSO. The absorbance of the solutions was determined spectrophotometrically at 570 nm with 660 nm as a reference using a plate reader (Victor³, PerkinElmer, Waltham, MA, USA). The absorbance of the control cells was taken as 100% viability. Experiments were performed in at least three independent replicates.

### Immunoblotting

Cells were treated for 3 h with increasing doses of ITCs (10, 20 and 40 µM SFN and 2.5, 5 and 10 µM PEITC, respectively) or were treated with 40 µM SFN or 10 µM PEITC for the indicated time. Cells treated with a vehicle (DMSO) served as a control. Cells were harvested, washed with ice-cold phosphate-buffered saline (PBS) and lysed on ice using a solution containing 50 mM Tris, 1% Triton X-100, 150 mM NaCl, 0.5 mM EDTA, protease and phosphatase inhibitor cocktails. The lysates were cleared by centrifugation at 15.500 g at 4℃ for 20 min. Proteins were resolved by SDS-polyacrylamide gel electrophoresis (SDS-PAGE) on 10% or 15% polyacrylamide gels. Electrophoresis was performed at a voltage of 60 V and 90 V during samples migration in the stacking and resolving gel, respectively. Next, proteins were transferred on a PVDF membrane by an overnight wet transfer at a voltage of 30 V at 4℃. The membrane was blocked with 5% non-fat dry milk in phosphate-buffered saline with 0.1% Tween-20 (PBST), and incubated with the desired primary antibodies overnight at 4 °C. After extensive washing with PBST, the membrane was incubated with appropriate secondary antibodies for 1 h at room temperature and washed again. The immunoreactive bands were detected with enhanced chemiluminescence reagent (Thermo Scientific Pierce, Rockford, IL, USA) on X-ray films (FujiFilm, Tokyo, Japan). The blots were stripped and reprobed with anti-β-actin or anti-GAPDH antibodies to normalize for differences in protein loading. The change in protein level was determined by densitometric analysis of the immunoreactive bands by Quantity One software (BioRad) followed by correction for the respective loading control (β-actin or GAPDH). The normalized value obtained in a control was taken as 1.0. Experiments were performed in at least three independent replicates.

### Protein synthesis assay

Cells were cultured in 12-well plates and treated for 3 h with a vehicle (DMSO; control), SFN (10, 20, 40 µM) or PEITC (2.5, 5, 10 µM) in the presence of 2 µCi/well L-[3,4,5-^3^H]-leucine. Cells were collected, fixed in 5% TCA at room temperature for 30 min and washed with 5% TCA for 15 min. The acid-insoluble material was dissolved in 0.1 M KOH overnight at 4^°^C and aliquots were used to determine the radioactivity using a liquid scintillation counter (Beckman Coulter, Brea, CA, USA). Experiments were performed in at least three independent replicates.

### Transient transfection with plasmid DNA

HDFa cells were transfected with plasmid DNA encoding GFP-HttQ74 (exon 1 of huntingtin with 74 polyglutamine residues, fused with EGFP) using Viromer Yellow transfection reagent (Lipocalyx GmbH, Halle, Germany) according to the manufacturer’s instruction. 48–72 h after transfection the cells were treated with a vehicle (DMSO; control), 40 µM SFN or 10 µM PEITC for 16 h and proceeded with the immunoblotting or fluorescence microscopy procedure. Experiments were performed in at least two independent replicates.

### Fluorescence microscopy

Cells were transfected with plasmid DNA encoding GFP-HttQ74 (exon 1 of huntingtin with 74 polyglutamine residues, fused with EGFP) and treated with isothiocyanates or a vehicle as described above. After the end of incubation, cells were washed with warm phosphate-buffered saline (PBS) and fixed with 2% paraformaldehyde in PBS (37^°^C) for 15 min. After washing with PBS, cells were counterstained with DAPI and washed again. Coverslips were mounted using Fluoromount mounting medium (Sigma Aldrich, St. Louis, MO, USA). The amount of GFP-tagged mHTT aggregates were examined with a fluorescent microscope (DMI4000B, Leica Microsystems GmbH, Wetzlar, Germany) with an appropriate filter set (Ex: 450–490 nm/Em: 500–550 nm). Experiments were performed in two independent replicates.

### Statistical analysis

Data were analyzed using the GraphPad Prism software. One-way ANOVA followed by Dunnett’s or Bonferroni’s multiple comparison tests was used to determine the statistical significance of the differences in the measured variables between control and tested groups. The difference was considered significant at p˂0.05. Symbols used indicate: * - *p* < 0.05; ** - *p* < 0.01; *** - *p* < 0.001.

## Results

### ITCs induce autophagy in primary human fibroblasts

**Fig. 1:**
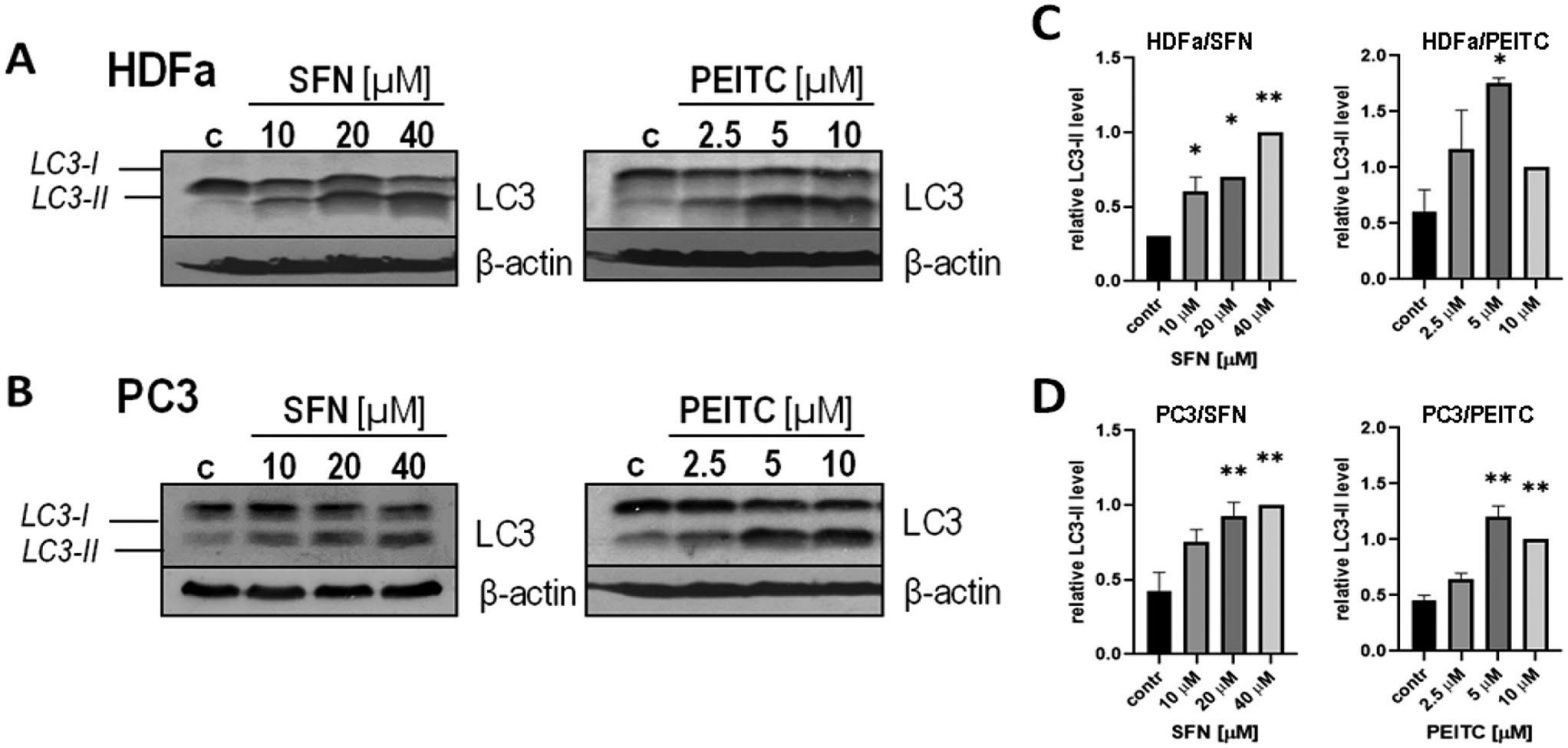
ITCs induce autophagy in both primary human fibroblasts and prostate cancer cells. Primary dermal fibroblasts (HDFa; **A,C**) and prostate cancer cells (PC3; **B, D**) were treated with the indicated concentration of SFN or PEITC for 3 h. Control cells (**c**) were treated with vehicle (DMSO). The cells were harvested and analyzed by immunoblotting to determine LC3-II (a marker of autophagy) and β-actin levels (control of equal protein loading). Graphs (**C-D**) show densitometric analysis corrected by protein loading (mean ± SEM). *, ** - significantly different (*p* < 0.05; *p* < 0.01, respectively) compared to control by one-way ANOVA followed by Dunnett’s Multiple Comparison Test

The growing number of evidence demonstrates that in cancer cells ITCs - including sulforaphane (SFN) and phenethyl isothiocyanate (PEITC) - efficiently induce autophagy [9, 15, 19–21]. However, their impact on primary cells, particularly of non-epithelial origin, is much less explored. Since malignant cells exhibit severe abnormalities in the control of crucial cellular processes, we raised the question of whether autophagy induction in response to ITCs is restricted to cancer cells or is a hallmark of healthy cells as well, particularly since data demonstrating such an effect in non-cancerous cells are limited. Moreover, the majority of such research has been conducted on immortalized cells of epithelial origin. Since immortalization obviously affects cell physiology by altering its proliferative capacity, it cannot be excluded that it might also affect the regulation of other cellular processes. Thus, to expand our knowledge, in our studies we decided to apply primary cells of mesenchymal origin, whose cellular processes are not affected by immortalization. For this purpose, we took advantage of adult-derived human dermal fibroblasts (HDFa) and exposed them to increasing doses of tested ITCs. As a reference, we used PC3 prostate cancer cells which are well-documented cellular models in ITCs-focusing studies [9, 15, 16, 19]. Cells of both cell lines were treated for 3 h with 10, 20 and 40 µM SFN or with 2.5, 5 and 10 µM PEITC. Control cells (c; contr) were treated with an equal volume of a vehicle, DMSO. Obtained results demonstrate that despite significant differences between tested cell lines (PC3 – epithelial, prostatic, cancerous, near-triploid with a modal number of 62 chromosomes; HDFa – mesenchymal, dermal fibroblasts, primary, diploid) both tested ITCs, SFN and PEITC, induced autophagy in primary cells to a similar extend as they did in malignant cells, as assessed by the level of LC3-II protein – a well-characterized autophagy marker (Fig. 1). The increase in LC-II level was dose-dependent in both cell lines.

### ITCs-induced autophagy does not affect the viability of primary cells

**Fig. 2:**
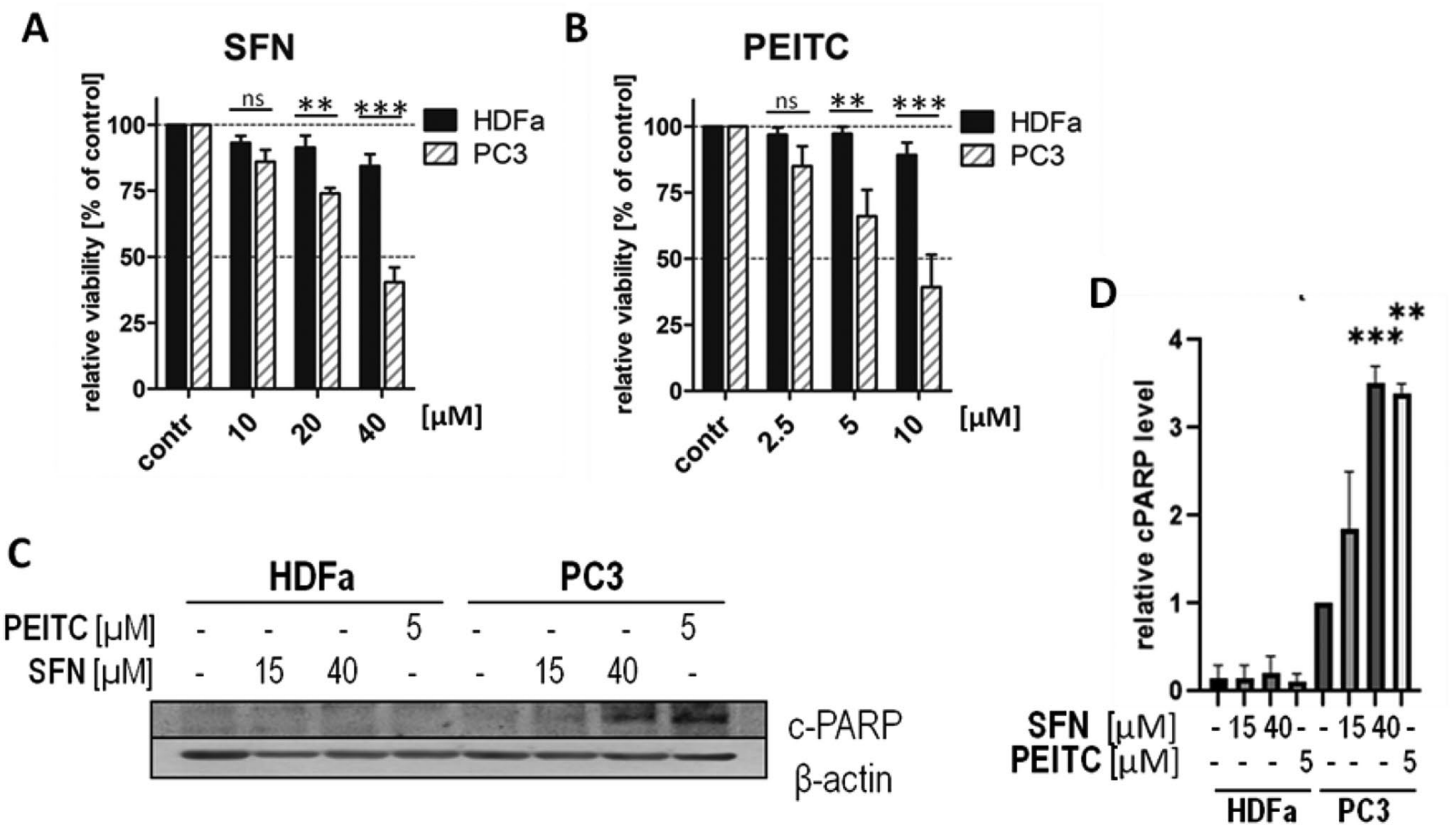
ITCs-induced autophagy neither affects viability nor induces apoptosis in primary cells. Primary dermal fibroblasts (HDFa) and prostate cancer cells (PC3) were treated with the indicated concentration of SFN or PEITC for 24 h (**A-B**) or 16 h (**C**). Control cells (contr) were treated with vehicle (DMSO). (**A-B**). Impact of SFN (**A**) or PEITC (**B**) on cell viability assessed by MTT test. Shown are mean ± SEM; **, *** - significantly different (*p* < 0.01; *p* < 0.001, respectively) by one-way ANOVA followed by Bonferroni’s Multiple Comparison Test; ns – not statistically significant. (**C**). Immunoblotting of cleaved PARP protein (cPARP; a marker of apoptosis) and β-actin (control of equal protein loading). A graph (**D**) shows densitometric analysis corrected by protein loading (mean ± SEM). **, *** - significantly different (*p* < 0.01; *p* < 0.001, respectively) compared to PC3 control by one-way ANOVA followed by Dunnett’s Multiple Comparison Test

Since autophagy is often activated during cell stress, we investigated whether ITCs treatment affects the viability of primary fibroblasts. As a reference, we applied prostate cancer cells (PC3). As expected, after 24-h treatment ITCs significantly and dose-dependently decreased the PC3 cells viability which lowered to about 40% for both, 40 µM SFN and 10 µM PEITC. However, ITCs only marginally affected the viability of fibroblasts, which was 84% and 89% when treated with SFN and PEITC, respectively, at the highest tested concentration (Fig. 2A-B).

Previous data showed that SFN and PEITCs induce apoptosis in cancer cells [9, 15, 16, 22]. To examine whether apoptosis induction occurs also in ITCs-treated fibroblasts we measured a level of cleaved PARP, a general marker of this process. Although we observed an appearance of a cleaved form of PARP in both SFN- and PEITC-treated PC3 cells, as expected, a respective band was absent in analogically treated primary fibroblasts (Fig. 2C-D). The obtained results confirmed the selective cytotoxicity of ITCs toward cancer cells. Moreover, they demonstrate that ITCs-induced autophagy has no apoptotic effect on primary fibroblasts.

### Autophagy induction by ITCs coincides with the inhibition of mTORC1 activity and protein synthesis in fibroblasts

**Fig. 3:**
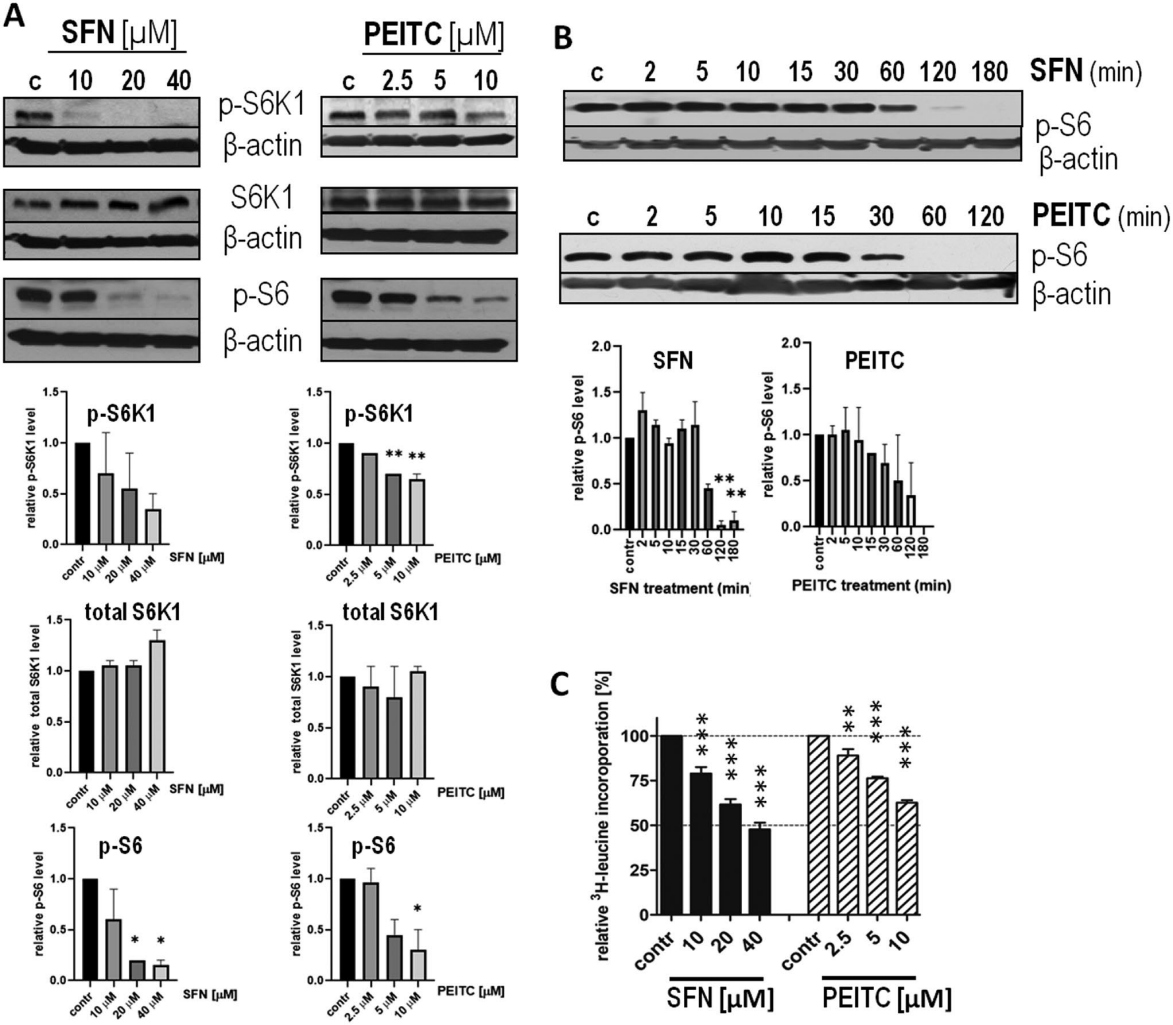
SFN and PEITC decrease the phosphorylation of mTORC1 substrates and inhibit protein synthesis in primary cells. Human dermal fibroblasts (HDFa) were treated with the indicated concentration of ITCs for 3 h (**A, C**) or with 40 µM SFN or 10 µM PEITC for the indicated time (**B**) (**A, B**). Immunoblotting of p-S6K1 (Thr-389), total S6K1, p-S6 (Ser-235), and β-actin (control of equal protein loading) in lysates from cells treated with different concentrations (**A**) or time (**B**) with ITCs. The graphs show a densitometric analysis of indicated proteins corrected by protein loading (mean ± SEM). *, ** - significantly different (*p* < 0.05; *p* < 0.01) compared to control by one-way ANOVA followed by Dunnett’s Multiple Comparison Test; (**C**). Relative [^3^H]–leucine incorporation in HDFa cells treated with the indicated concentration of ITC for 3 h in the presence of a protein precursor, [^3^H]–leucine. Shown are mean ± SEM; **, *** - significantly different (*p* < 0.01; *p* < 0.001, respectively) compared to control by one-way ANOVA followed by Dunnett’s Multiple Comparison Test

The main negative regulator of autophagy in a cell is mTORC1. When active, it promotes anabolic processes such as protein synthesis, lipogenesis and mitochondrial biogenesis and concomitantly inhibits autophagy [23]. However, cases of mTORC1-independent autophagy were also reported [24–26]. To verify whether the observed induction of autophagy by ITCs in fibroblasts is a result of mTORC1 inhibition, we measured the phosphorylation level of mTORC1 substrate, S6K1, at Thr-389 - modification essential for the activity of S6K1. In both, SFN- and PEITC-treated cells we observed a dose-dependent decrease in S6K1 phosphorylation (Fig. 3A). The total level of S6K1 remained constant confirming that decreased level of its phosphorylated form is not a result of S6K1 degradation. A drop in phosphorylation of S6K1 was followed by a decrease in phosphorylation of S6K1 substrate, ribosomal protein S6, confirming that S6K1 is not only dephosphorylated but also inactivated (Fig. 3A-B).

mTORC1-S6K1 is a signaling pathway primarily controlling several stages of mRNA translation. To confirm its inactivation by tested ITCs, we measured the rate of protein synthesis by assessing the incorporation of ^3^H-leucine into cellular proteins. As demonstrated in Fig. 3C, protein synthesis measured after 3-hour exposure to SFN or PEITC was inhibited in a dose-dependent manner. Incorporation of [^3^H]-leucine in HDFa cells exposed to 40 µM SFN decreased to 46% of the level seen in control cells, whereas in 10 µM PEITC-treated cells - to 66%. A block in protein synthesis together with a decreased phosphorylation of S6K1 and ribosomal S6 protein confirms inhibition of mTORC1 activity by SFN and PEITC in primary fibroblasts.

### mTORC1 inhibition by ITCs in primary cells is preceded by AMPK activation

**Fig. 4:**
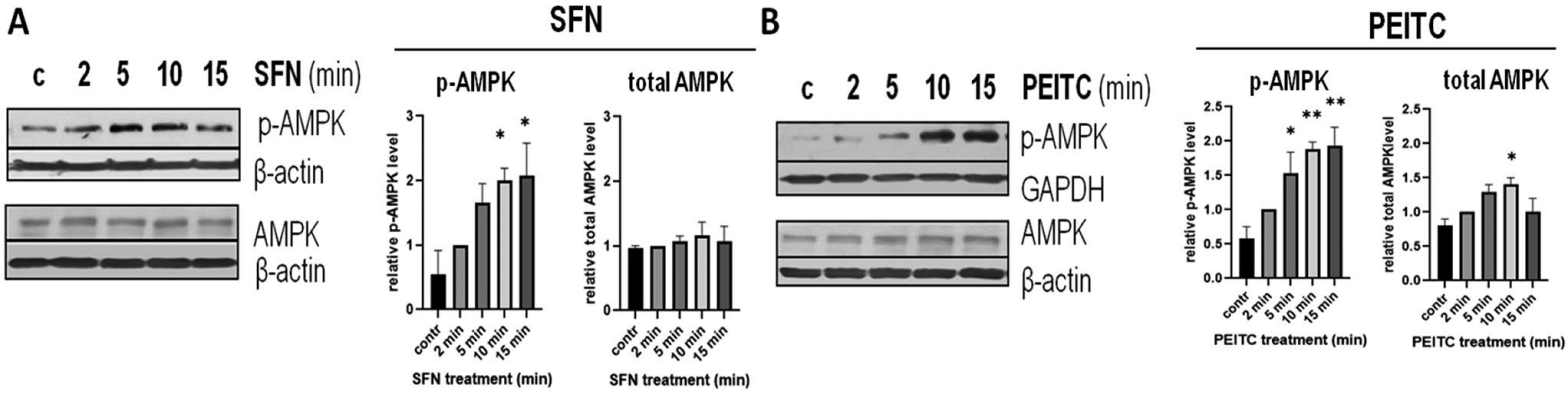
AMPK activation precedes mTORC1 inhibition in ITCs-treated primary human fibroblasts (HDFa). Cells were treated with 40 µM SFN (**A**) or 10 µM PEITC (**B**) for the indicated time. The amount of active, phosphorylated at Thr-172, form of AMPK was assessed by immunoblotting. The blots were stripped and reprobed with anti-total AMPK antibodies and anti-β-actin or anti-GAPDH antibodies to ensure equal protein loading. The graphs below show a densitometric analysis of indicated proteins corrected by protein loading (mean ± SEM). *, ** - significantly different (*p* < 0.05; *p* < 0.01, respectively) compared to control by one-way ANOVA followed by Dunnett’s Multiple Comparison Test

Because of its role in suppressing energy-consuming processes, AMPK is known as the physiological inhibitor of mTORC1/S6K1 signaling. This prompted us to investigate whether mTORC1 inhibition observed in cells treated with ITCs results from AMPK activation. To address this issue, we measured phosphorylation of AMPK at Thr-172, which is a marker of AMPK activity, at time points prior to a drop in S6 protein phosphorylation which was observed within 30–60 min after exposure to ITCs (Fig. 3B). As shown in Fig. 4, exposure of fibroblasts to SFN or PEITC resulted in an increase in AMPK phosphorylation indicating its activation. An upregulation of phospho-AMPK was observed as soon as 2–5 min after the addition of SFN and 5 min after the addition of PEITC, thus, it preceded inhibition of S6 phosphorylation and might be its cause.

### PEITC-induced protein synthesis block, mTORC1 inhibition and AMPK activation are not restricted to fibroblasts

**Fig. 5:**
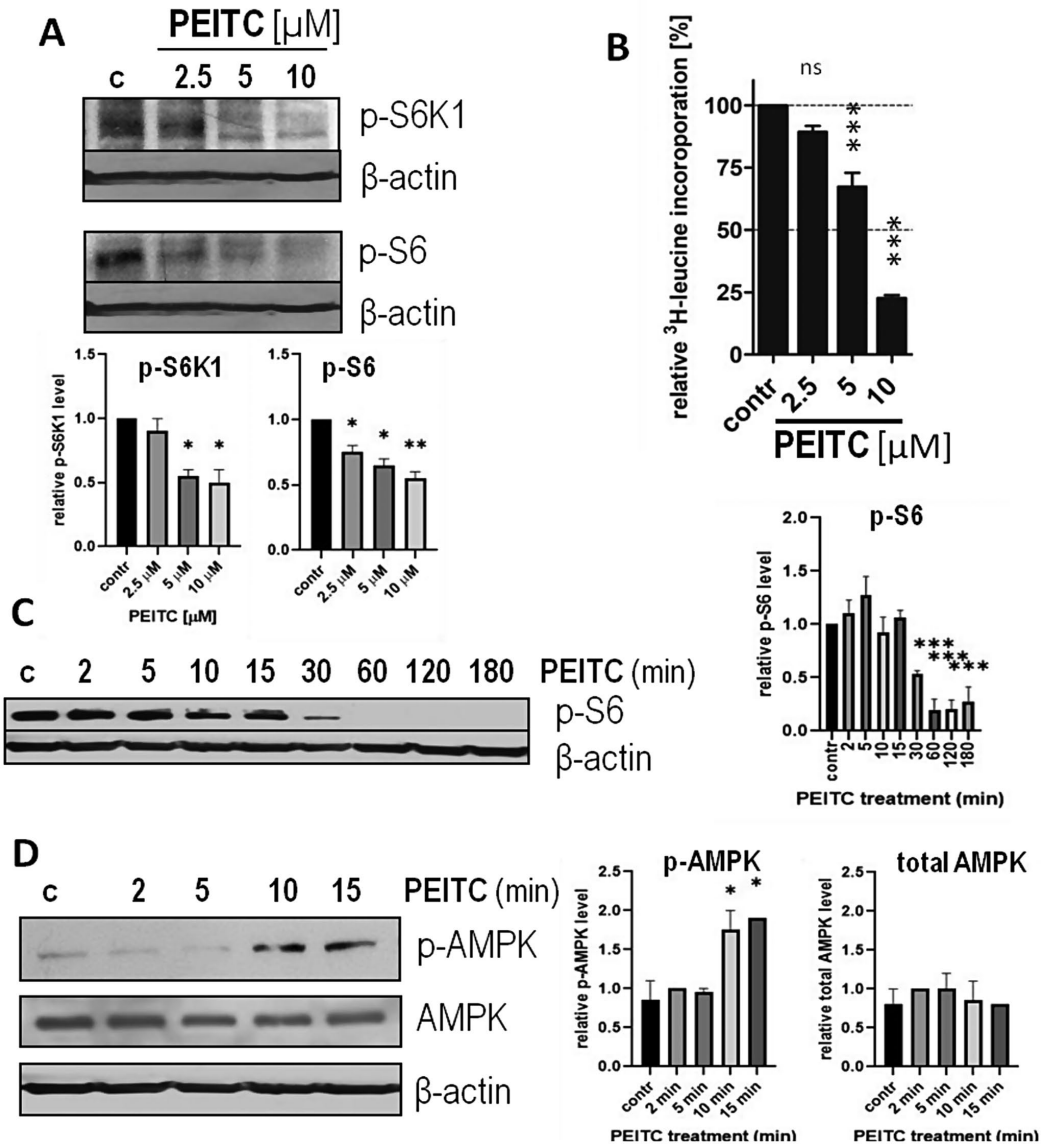
PEITC induces protein synthesis block, mTORC1 inhibition and AMPK activation in prostate cancer (PC3) cells. PC3 cells were treated with the indicated concentrations of PEITC for 3 h (**A-B**) or with 10 µM PEITC for the indicated time (**C-D**). Control cells (c; contr) were treated with vehicle (DMSO) (**A, C**). Immunoblottings of p-S6K1 (Thr-389), p-S6 (Ser-235), and β-actin (control of equal protein loading) in lysates from cells treated with different concentration (**A**) or time (**C**) with PEITC. Graphs below (**A**) or on the right (**C**) show densitometric analysis of indicated proteins corrected by protein loading (mean ± SEM). *, **, *** - significantly different (*p* < 0.05; *p* < 0.01; *p* < 0.001, respectively) compared to control by one-way ANOVA followed by Dunnett’s Multiple Comparison Test; B. Relative [^3^H]–leucine incorporation in PC3 cells treated with the indicated concentrations of PEITC for 3 h in the presence of a protein precursor, [^3^H]–leucine. Shown are mean ± SEM; *** - significantly different (*p* < 0.001) compared with control by one-way ANOVA followed by Dunnett’s Multiple Comparison Test; (**D**). The amount of active, phosphorylated at The-172, form of AMPK was assessed by immunoblotting. The blots were stripped and reprobed with anti-total AMPK antibodies and anti-β-actin antibodies to ensure equal protein loading. Graphs on the right show densitometric analysis of indicated proteins corrected by protein loading (mean ± SEM). * - significantly different (*p* < 0.05) compared to control by one-way ANOVA followed by Dunnett’s Multiple Comparison Test

To determine whether PEITC-induced protein synthesis block, mTORC1 inhibition and AMPK activation are limited to fibroblasts or are a more general phenomenon, were performed parallel experiments on prostate cancer cells (PC3). In accordance with previous results [15], in PC3 cells treated with increasing doses of PEITC, mTORC1 activity was inhibited as detected by a decrease in the phosphorylation of S6K1 at Thr-389 and its substrate, S6 ribosomal protein (Fig. 5A). It also potently inhibited translation as measured by a [^3^H]-leucine incorporation − 10 µM PEITC caused a drop in protein synthesis to 21% of the level in control cells (Fig. 5B). In a time-course experiment, treatment of PC3 cells with PEITC resulted in a decreased phosphorylation of S6, which was observed after 10 min of exposure (Fig. 5C). Similarly as in primary cells, this was preceded by the activation of APMK, which phosphorylation increased 10 min after exposure to PEITC (Fig. 5D).

### ITCs decrease the amount of mutant huntingtin (mHtt) aggregates in a cellular model of Huntington’s disease

**Fig. 6:**
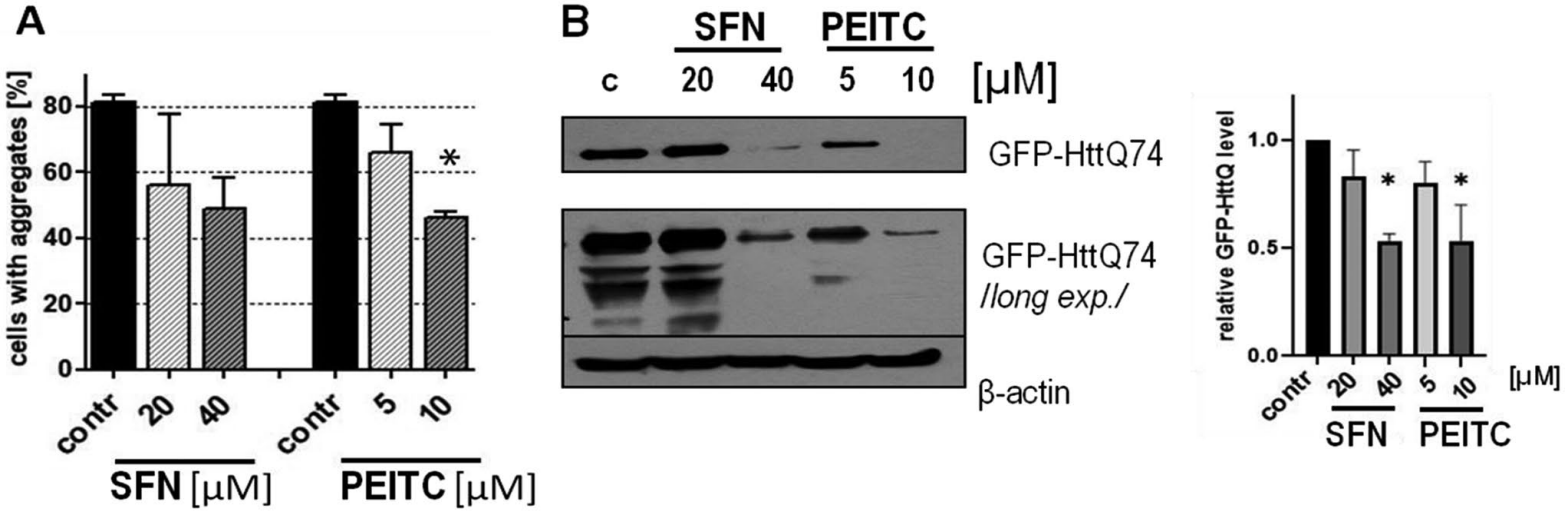
ITCs decrease the amount of mutant huntingtin (mHtt) protein and its aggregates in primary cells. Primary human fibroblasts (HDFa) were transfected with the vector encoding mutated exon 1 of huntingtin fused with GFP (mHtt-GFP). 48–72 h post transfection cells were treated with vehicle (control; c), 20 or 40 µM SFN, 5 or 10 µM PEITC for 16 h. (**A**) The number of cells with aggregates (% of the total cell number) was evaluated by fluorescence microscopy. Shown are mean ± SEM; * - significantly different (*p* < 0.05) compared to control by one-way ANOVA followed by Dunnett’s Multiple Comparison Test. (**B**) Total mHtt-GFP level in cellular lysates was assessed by immunoblotting with anti-GFP antibodies. Additional bands are visible after prolonged exposure and represent products of partial mHtt-GFP degradation. The blots were stripped and reprobed with anti-β-actin antibodies to ensure equal protein loading. The graph below shows a densitometric analysis of GFP-HttQ74 corrected by protein loading (mean ± SEM). * - significantly different (*p* < 0.05) compared to control by one-way ANOVA followed by Dunnett’s Multiple Comparison Test

Autophagy was shown to be involved in the efficient removal of protein aggregates [27, 28]. Stress induced by protein aggregation can have deleterious consequences for a cell and underlies a pathomechanism of a group of neurodegeneration diseases (proteinopathies) and other disorders [4, 29]. This prompted us to explore whether ITCs could affect the amount of mutant huntingtin (mHtt) aggregates – a major cause of Huntington’s disease. For this purpose, we transfected fibroblasts with a vector encoding exon 1 of mutant huntingtin (mHtt) fused with GFP. Obtained results demonstrated that both ITCs, SFN and PEITC, decreased the number of cells with intracellular mHtt aggregates from 81% observed in control cells to 56% and 49% in cells treated with 20 µM or 40 µM SFN, respectively, and to 66% and 46% for cells treated with 5 µM or 10 µM PEITC, respectively (Fig. 6A).

To assess the impact of ITCs on aggregates morphology, i.e. their number and size, we created a four-class scale: 0 – cells without visible aggregates; I – 1–3 aggregates per cell; II - numerous small aggregates per cell; III – cells with large aggregates (Supplemental Fig. S1A). After categorizing cells into appropriate classes, we observed that within the control sample (treated with a vehicle) cells possessing mHtt aggregates represented all three classes of aggregation (i.e. I-III). However, in ITCs-treated cells, there was a noticeable drop in the number of cells in I, II and III classes, and among cells with aggregates, the dominant fraction constituted the cells with only 1–3 aggregates per cell (Class I) (Supplemental Fig. S1B-C).

To verify our results, we determined the impact of ITCs on the total level of mHtt in cells by immunoblotting. The results presented in Fig. 6B clearly showed that both tested ITCs efficiently decreased the amount of mHtt in cells.

Altogether, these findings indicate that ITCs are efficient agents blocking the formation of mutant huntingtin aggregates, which are the main cause of its toxicity, and thus might have a potential in developing therapeutics for Huntington’s disease and, perhaps, other proteinopathies.

## Discussion

Our results revealed that both tested isothiocyanates, SFN and PEITC, induce autophagy in primary (and thus non-transformed and non-immortalized) mesenchymal cells as potently as in cancer cells. In primary cells, ITCs-induced autophagy was mediated by activation of AMPK that inhibited mTORC1, thus releasing autophagy from its inhibitory effect and blocking protein synthesis. This seems an universal mechanism as AMPK activation, mTORC1 inhibition, translation block and autophagy stimulation we also observed in PEITC-treated prostate cancer cells (PC3). Moreover, these results are consistent with previous reports showing autophagy induction and mTORC1 inhibition in breast and prostate cancer cells with different genetic backgrounds or treated with another ITC, benzyl isothiocyanate (BITC) [9, 15, 19, 20, 30]. However, despite the potent induction of autophagy, ITCs did not affect the viability of primary cells and exert no apoptotic effect.

Presented results indicated that tested ITCs, SFN and PEITC, mimic two phenomena induced by caloric restriction, namely a reduction of protein synthesis coupled to an increase in autophagy, which occurs via AMPK-mTORC1-S6K1 pathway, which may underlie at least some of the health-promoting properties of isothiocyanates. Both autophagy induction and AMPK activation possess great therapeutic potential. Stimulation of autophagy (and mTORC1 inhibition) and of AMPK activity are potential mediators of the beneficial effects of caloric restriction and physical exercise on human health as well as of improvement of metabolic conditions in patients suffering from type 2 diabetes, obesity and cardiovascular diseases [10–12, 31–33]. Nowadays obesity has reached a level of epidemic proportions worldwide and is considered a disease of the 21st century civilization. Numerous studies indicate that obesity and weight gain are associated with an increased risk of developing insulin resistance, diabetes and heart disease [33, 34]. On the other hand, caloric restriction delays the onset of numerous age-associated diseases including cancer, atherosclerosis, and diabetes, and can greatly increase a life span [10, 32]. At the molecular level, this beneficial effect is also attributed to autophagy induction, mTORC1 inhibition and AMPK activation [11, 12]. AMPK was shown to be activated by several natural agents with beneficial effects for human health including resveratrol, quercetin, ginsenoside or curcumin [32, 33, 35, 36]. Therefore, it is possible that ITCs-mediated activation of AMPK and autophagy would mimic the effects of caloric restriction and exercise in humans, and thus could be applied as a potential therapeutics for metabolic syndrome, including type 2 diabetes and obesity, as recently suggested in research with obese mouse model and sulforaphane treatment [37, 38].

One of the main issue for further investigation is the molecular mechanism of AMPK activation by ITCs. It was reported that AMPK can be activated in response to various stimuli including changes in AMP/ATP ratio, Ca^2+^ level, DNA damage and increased ROS generation [39–42]. Our previous research performed on prostate cancer cells (PC3) treated with SFN indicated that there was no ATP level drop in cells (19). In addition, our recently published data revealed that there was no genotoxic stress in SFN and PEITC-treated fibroblasts [43]. It is possible that ITCs activate AMPK by an increase in reactive oxygen species (ROS) as was observed in cancer cells [19, 44]. However, in cancer cells the elimination of mitochondria-derived ROS only partially protected against autophagy induction which suggests that another mechanism contributes to AMPK activation and autophagy induction. Therefore, the molecular mechanism leading to AMPK activation by ITCs still needs to be deciphered. However, it is worth noting that despite autophagy induction, tested ITCs do not exert a detrimental impact on primary cells viability, in contrast to cancer cells (Fig. 2).

Autophagy plays an important role particularly during cell stress, enabling, among others, the elimination of damaged organelles or protein aggregates [27, 28]. Stress induced by cellular protein aggregates can have deleterious consequences for the cell, contributing to neurodegeneration, including Huntington’s disease and Alzheimer’s disease, as well as other disorders [2–5, 29, 45]. Currently, there is no effective method to prevent or slow down the progression of neurodegenerative diseases. In 2002, it was proposed that activation of autophagy could help to prevent the accumulation of toxic protein aggregates [4]. Studies performed on cellular as well as *Drosophila* and a mouse model of Huntington’s disease showed that the induction of autophagy by specific inhibitors of mTORC1 complex, as the major negative regulator of autophagy in cells, reduced the amount of mutant huntingtin aggregates and their cytotoxicity [4]. Inhibition of autophagy had the opposite effect [4, 46]. This phenomenon was not limited to mutant huntingtin. In the model of Alzheimer’s disease, aggregation-prone protein - amyloid β (Aβ) - was also removed via autophagy [47]. Proper progress of this process appears to be crucial for the prevention of neurodegeneration, even in the absence of mutant proteins in a cell. Studies performed on autophagy-defective mice have shown that at a cell level, the absence of autophagy resulted in the accumulation of ubiquitin, formation of protein aggregates and induction of reticular stress, leading to the development of the phenotype characteristic for neurodegeneration at the organismal level. A similar pattern of intracellular changes was observed in fibroblasts during the formation of fibrosis [5, 48, 49]. Therefore, the strategy of intensifying autophagy might be a potential therapeutic approach enabling the reduction of the accumulation of toxic protein aggregates in cells. Our results demonstrated that both SFN and PEITC efficiently decreased the number of mutant huntingtin aggregates in cells. This effect may result from potent induction of autophagy and concomitant inhibition of protein synthesis. As ITCs were shown to be safe and do not cause side effects in animal models [18], they might have a potential in developing therapeutics for Huntington’s disease and, possibly, other proteinopathies.

The aim of presented studies was to compare whether the effects exerted by SFN and PEITC in cancer cells (including autophagy induction) are also observed in primary non-transformed cells. For this purpose, in our research we used the same concentration of SFN and PEITC (representing their IC50 value) as in previously published studies performed on PC3 prostate cancer cells [9, 15, 16, 22, 50]. The key issue is whether such concentrations are achievable in vivo. Pharmacokinetic studies in both humans and animals show that SFN and PEITC can achieve micromolar concentrations in plasma and accumulate in tissues. This process is rapid, with peak plasma concentration between 1 and 4 h after administration of ITCs [51, 52]. In rats a single oral dose of 50 µmol of SFN leaded to a peak plasma concentration around 20 µM whereas oral doses of 10 and 100 µmol/kg of PEITC to a peak around 10 and 40 µM, respectively [52, 53]. Accumulation of dietary ITCs in human cells and tissues in vivo is still not fully investigated. Pharmacokinetic studies conducted on a group of 4 volunteers receiving broccoli sprout extract containing approximately 200 mmol total ITCs mixture (including SFN) showed that ITCs were absorbed rapidly and reached peak concentrations of ~ 1 µM in plasma 1 h after ingestion of the extract [54]. A similar peak plasma concentration of PEITC (~ 1 µM) was noted in volunteers who received a single dose of 100 g watercress (containing ~ 153 µmol PEITC) [55]. However, since in these studies humans were not treated with pure ITCs but the broccoli sprouts extract or watercress, it cannot be excluded that a presence of other active substances affect ITCs bioavailability. Pharmacokinetic studies using pure ITCs are needed to answer to this question. The picture is even more complicated as previous data demonstrated that ITCs (including SFN and PEITC) can accumulate in mammalian cells in up to millimolar concentrations [56]. Thus, the circulating plasma level of ITCs might be affected by this process.

The ability to go through the blood-brain barrier (BBB) is essential to exert neuroprotective effects for any phytochemicals. An animal studies on mice shown SFN to cross the BBB and accumulate at nmolar concentrations in brain tissue after a gavage of either 5 or 20 µmoles of SFN [57, 58]. The studies of Jazwa et al. demonstrated that SFN accumulate in cerebral tissues such as the ventral midbrain and striatum after intraperitoneal injection of 50 mg/kg SFN and repeated administration of SFN was sufficient to protect against MPTP-induced death of nigral dopaminergic neurons [59]. As most tissues followed similar kinetics as plasma, with highest concentrations at 2 h and complete clearance by 24 h, it suggest that repeated administration of SFN might be required for keeping an effective SFN concentration in the brain. In a context of our research, as a next step, it will be worth investigating whether the administration of lower doses of ITC but repeatedly for a longer period of time would be able to exert a similar, desired effect in terms of induction of autophagy and prevention of abnormal Htt aggregates formation.

Several transgenic and knock-in rodent models have been generated to investigate pathological features of Huntington’s disease (HD) [60]. However, significant species differences between rodent and human cells limit the use of HD rodent models to accurately represent the disease [61]. An alternative is reprogramming of HD patient-derived fibroblasts into iPSC to generate human striatal neurons carrying the huntingtin mutation. They are a powerful tool to investigate this genetic disorders, however, the limitations of this model are a low efficiency and reproducibility of published protocols, a high heterogeneity of obtained neurons and clone-to-clone variability [61, 62]. Moreover, by passing through a pluripotent state, neurons lose the majority of the epigenetic information inherent in cells of a particular donor. Another option is a direct reprogramming of HD patient-derived fibroblasts into striatal neurons. Since this approach omits embryonic state, yields neurons that preserve the epigenetic information of a particular donor and, consequently, the age-associated disease phenotype [62].

Although Huntington’s disease is a hereditary neurodegenerative disorder caused by preferential neuronal death in specific brain regions, in our cellular model of this disease we decided to use fibroblasts as a host for transfection with a plasmid encoding mutant huntingtin (mHtt). First, we wanted to keep to the model of primary cells, non-transformed neoplastically nor immortalized. In studies on neurodegenerative disorders (including Huntington’s disease) commonly used are neuronal cell lines derived from neuroblastomas (e.g. mouse Neuro-2a, human SK-N-MC and SH-SY5Y) or rat pheochromocytoma (PC12), which can be differentiated into neurons. However, we wanted to avoid neoplastic cell lines since the aim of the presented studies was to examine the response of primary cells (with a normal karyotype, undergoing natural telomerase-dependent senescence etc.). Moreover, although the use of transfected dermal fibroblasts does not reflect the neuronal aspect of Huntington’s disease, on the other hand, numerous reports described - in addition to changes in the CNS - additional abnormalities in the peripheral tissues of HD patients, including accumulation of intracellular protein aggregates, altered glucose homeostasis, transcriptional deregulation and sub-cellular abnormalities in fibroblasts, lymphocytes and erythrocytes [63–66]. It was also suggested that the molecular mechanisms through which mHtt leads to cell dysfunction are widely shared between the central nervous system (CNS) and peripheral tissues [63, 66]. Thus, molecular changes detected in transfected fibroblasts treated with ITCs might be a model reflecting processes induced in peripheral tissues. Thus, our results constitute a basis for broader investigation, particularly in more advanced models of neurons derived from HD patient fibroblasts and in animal models.

## Conclusions

We demonstrate that isothiocyanates (SFN and PEITC) induce autophagy in primary cells as potently as in cancer cells and via a similar molecular mechanism, i.e. modulation of the AMPK-mTORC1-S6K1 pathway, which results in the block of protein synthesis and induction of autophagy. Moreover, since ITCs did not affect the viability of primary cells, their ability to induce autophagy and block protein synthesis can be applied to protect a cell against the accumulation of harmful protein aggregates of mutant huntingtin (mHtt) and to decrease its total cellular level. Since the aggregates of this abnormal protein are a main cause of Huntington’s disease, it suggests that ITCs might have a potential in developing therapeutics for this disorder.

## Electronic supplementary material

Below is the link to the electronic supplementary material.

Supplementary Material 1

## Data Availability

The data supporting the findings of this study are available from the corresponding author upon reasonable request.
