# Supplementary material for: Isothiocyanates induce autophagy and inhibit protein synthesis in primary cells via modulation of AMPK-mTORC1-S6K1 signaling pathway, and protect against mutant huntingtin aggregation
Source: Eur J Nutr. 2024 Dec 16;64(1):46. doi: 10.1007/s00394-024-03539-z (PMC11649724; doi:10.1007/s00394-024-03539-z)
Supplement: Supplementary file 1 — Supplementary Material 1 [file 394_2024_3539_MOESM1_ESM.pdf]

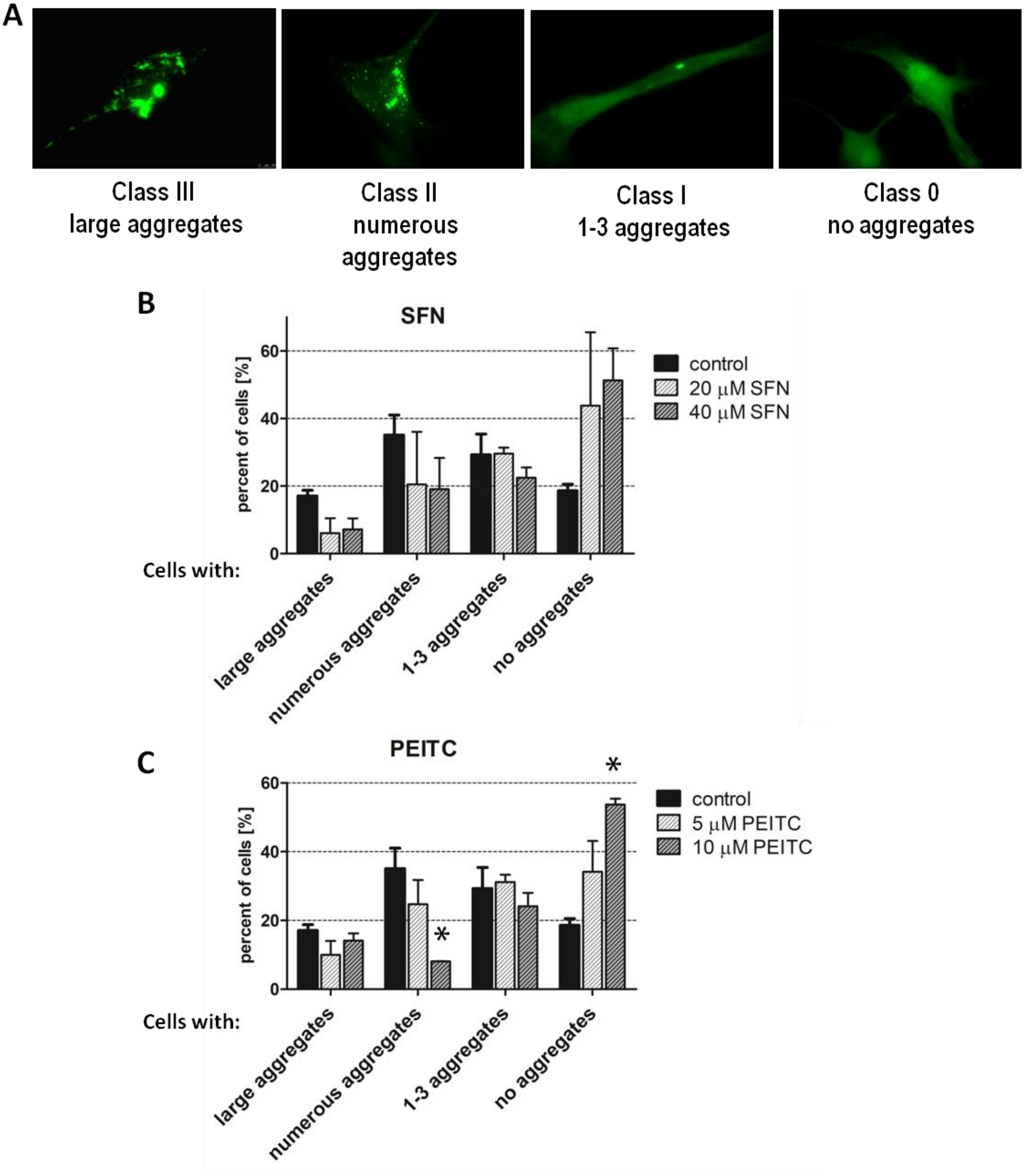

Supplementary Fig. S1 The impact of ITCs on the number and size of mutant huntingtin (mHtt-GFP) aggregates in normal cells Normal fibroblasts (HDFa) were transfected with vector encoding mutated exon 1 of huntingtin fused with GFP (mHtt-GFP). 48-72 h post transfection cells were treated with vehicle (control; DMSO), 20 or 40 µM SFN (B), 5 or 10 µM PEITC (C) for 16h. A. Representative images of cells categorized to different classes of aggregation depending on aggregates number and size; B, C. Relative distribution of control and ITCs-treated cells in different aggregation classes. Shown are mean ± SEM; * - significantly different ($p<0.05$) compared to control by one-way ANOVA followed by Bonferroni's Multiple Comparison.

"Isothiocyanates induce autophagy and inhibit protein synthesis in normal cells *via* modulation of AMPK-mTORC1-S6K1 signaling pathway, and protect against mutant huntingtin aggregation" *European Journal of Nutrition* Brokowska, J., Herman-Antosiewicz, A., Hać, A. Department of Medical Biology and Genetics, Faculty of Biology, University of Gdansk, Stwosza 59, 80-308 Gdansk, Poland; aleksandra.hac@ug.edu.pl
